# A machine learning-based prediction model for postoperative delirium in cardiac valve surgery using electronic health records

**DOI:** 10.1186/s12872-024-03723-3

**Published:** 2024-01-18

**Authors:** Qiuying Li, Jiaxin Li, Jiansong Chen, Xu Zhao, Jian Zhuang, Guoping Zhong, Yamin Song, Liming Lei

**Affiliations:** 1 Department of Cardiac Surgical Intensive Care Unit, Guangdong Cardiovascular Institute, Guangdong Provincial People’s Hospital, Guangdong Academy of Medical Sciences, Southern Medical University, Guangzhou, 510080 China; 2 Department of Cardiovascular Surgery, Guangdong Cardiovascular Institute, Guangdong Provincial People’s Hospital, Guangdong Academy of Medical Sciences, Southern Medical University, Guangzhou, 510080 China; 3 Guangdong Provincial Key Laboratory of South China Structural Heart Disease, Guangdong Cardiovascular Institute, Guangdong Provincial People’s Hospital, Guangdong Academy of Medical Sciences, Southern Medical University, Guangzhou, 510080 China; 4 Institute of Clinical Pharmacology, Guangdong Provincial Key Laboratory of New Drug Design and Evaluation, School of Pharmaceutical Sciences, Sun Yat-Sen University, Guangzhou, Guangdong China; 5 Shantou University Medical College (SUMC), Shantou, 515041 China; 6 Department of Cardiovascular Surgery, Guangdong General Hospital’s Nanhai Hospital, The Second People’s Hospital of Nanhai District, Foshan, Guangdong 528251 China

**Keywords:** Prediction model, Postoperative delirium, Random Forest Classifier, Machine learning, Cardiac valve surgery

## Abstract

**Background:**

Previous models for predicting delirium after cardiac surgery remained inadequate. This study aimed to develop and validate a machine learning-based prediction model for postoperative delirium (POD) in cardiac valve surgery patients.

**Methods:**

The electronic medical information of the cardiac surgical intensive care unit (CSICU) was extracted from a tertiary and major referral hospital in southern China over 1 year, from June 2019 to June 2020. A total of 507 patients admitted to the CSICU after cardiac valve surgery were included in this study. Seven classical machine learning algorithms (Random Forest Classifier, Logistic Regression, Support Vector Machine Classifier, K-nearest Neighbors Classifier, Gaussian Naive Bayes, Gradient Boosting Decision Tree, and Perceptron.) were used to develop delirium prediction models under full (q = 31) and selected (q = 19) feature sets, respectively.

**Result:**

The Random Forest classifier performs exceptionally well in both feature datasets, with an Area Under the Curve (AUC) of 0.92 for the full feature dataset and an AUC of 0.86 for the selected feature dataset. Additionally, it achieves a relatively lower Expected Calibration Error (ECE) and the highest Average Precision (AP), with an AP of 0.80 for the full feature dataset and an AP of 0.73 for the selected feature dataset. To further evaluate the best-performing Random Forest classifier, SHAP (Shapley Additive Explanations) was used, and the importance matrix plot, scatter plots, and summary plots were generated.

**Conclusions:**

We established machine learning-based prediction models to predict POD in patients undergoing cardiac valve surgery. The random forest model has the best predictive performance in prediction and can help improve the prognosis of patients with POD.

## Introduction

Postoperative delirium (POD) is a series of acute and paroxysmal neurocognitive disorders after cardiac surgery. Symptoms include inattention, disorganized thinking, and altered states of consciousness, which are not attributable to other known psychiatric conditions or neurological disorders [1–3]. In addition, POD occurs in three forms: hyperactive, hypoactive, and mixed delirium, which are often difficult to diagnose. The pathogenesis of POD remains unclear, and there are currently no effective diagnostic tools available to distinguish between ordinary agitation and POD. POD has been associated with increased mortality, prolonged hospitalization, long-term cognitive dysfunction, impaired quality of life, and increased healthcare costs [4–7]. As a result, healthcare providers and policymakers have recommended that POD prediction models be used at various stages of the clinical pathway to support decision-making [8].

Although ICU clinicians have focused on delirium in patients after cardiac surgery with cardiopulmonary bypass (CPB) as a unique contributor to neurocognitive dysfunction [6, 9, 10], current studies of prediction models often lump all cardiac surgeries together, ignoring the potential influence of cardiac disease and surgical modalities on the onset of delirium. This study focused mainly on the occurrence of delirium in VHD patients after cardiac surgery with CPB, as the number of such surgeries has increased over the past decades. Valve replacement or repair is the first option for VHD [11]. Early diagnosis methods have been developed to facilitate earlier valve replacement or repair in VHD patients. The increasing number of surgeries associated with VHD is attributed to its increasing incidence due to the aging population worldwide [12, 13]. Advanced age has been identified as a risk factor for delirium [7, 10, 14], and the prediction and management of delirium is particularly significant in the VHD surgical population.

An effective POD prediction model can greatly assist ICU clinicians in predicting patients at high risk of developing POD. This information can then be used to create better treatment plans and care protocols to help prevent the onset of delirium. However, few existing predictive models use machine learning algorithms, and many of these models have a high risk of bias [14–17]. We aim to develop and validate a prediction model using machine learning tools that adhere to the standards set by the Transparent Reporting of a Multivariable Prediction Model for Individual Prognosis or Diagnosis (TRIPOD) statement: a guideline specifically designed to guide the reporting of studies that create or validate multivariable prediction models [18].

## Materials and methods

### Study population

**Fig. 1:**
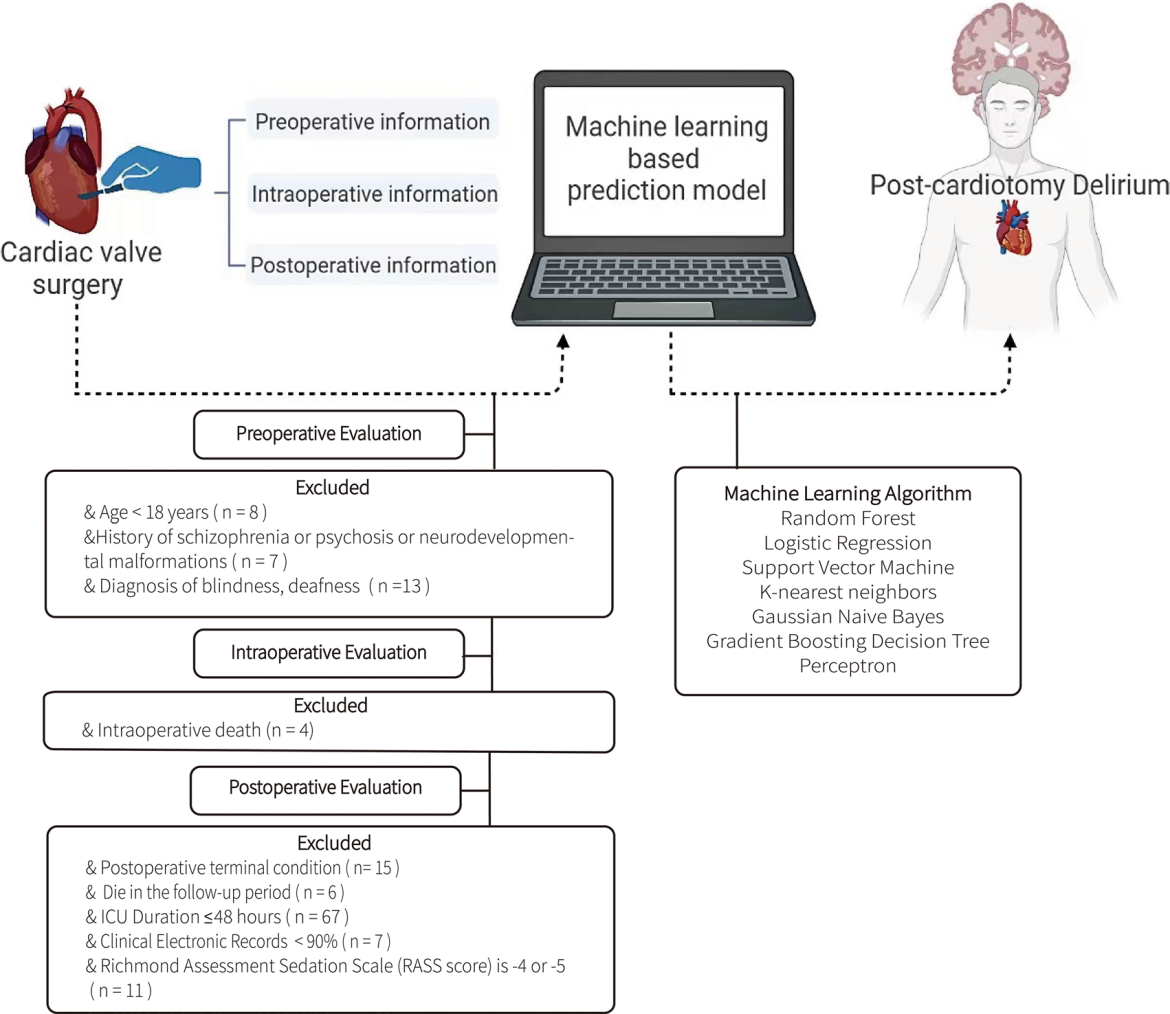
The screening process for patient enrollment in this study

Data on clinical characteristics and outcomes of patients with VHD who underwent cardiac valve surgery with CPB were collected from the computerized database of the CSICU of Guangdong Provincial People’s Hospital. The screening process for cases entering the group is shown in Fig. 1. Referring to previous studies, we have established the following inclusion criteria in this study [14, 19–21]: (I) over 18 years of age; (II) definite diagnosis of valvular heart disease; (III) admission to the CSICU after cardiac valve surgery with CPB; (IV) no history of schizophrenia, psychosis, or neurodevelopmental malformations; (V) no diagnosis of blindness, deafness, or drug abuse or withdrawal; (VI) not in a terminal condition with an ICU stay more than 48 h; (VII) delirium as assessed by trained paramedics using the Confusion Assessment Method for the ICU (CAM-ICU) and the Richmond Assessment Sedation Scale (RASS) score of − 3 to + 4; (VIII) no reoperation during the follow-up period; and (IX) clinical records completed at least 90%. Our study comprehensively addresses the ethical, legal, and regulatory norms and standards for conducting research involving clinical data in China, including relevant international norms and standards. Throughout the data collection phase, strict measures are implemented to protect privacy, ensuring that all information is anonymized.

### Selected variables

This study identified three categories (preoperative, intraoperative, and postoperative) and 31 potential risk factors for delirium based on previous research and the availability of clinical records in our electronic database. They include demographic characteristics, lifestyle factors, cognitive function, physical function, social-psychological factors, sensory function, pre-existing diseases, surgical information, and postoperative laboratory test indicators [22]. Our sample size meets the events per variable (EPV) criteria, which ensures the reliability and effectiveness of statistical analysis [23].

In addition to the full feature set with 31 potential risk factors, we have also identified a simple feature set consisting of 19 predictor variables. These variables have a lower likelihood of missing data and exhibit a strong correlation with delirium. They are referred to as the selected feature set. The selection of features in the simple set was based on existing literature and ease of data collection, aiming to provide valuable insights for clinicians in making clinical judgments when examination results are insufficient. The process of selecting features was conducted meticulously, involving a thorough review by a panel of experts in the fields of cardiac surgery, delirium, anesthesiology, neurology, cardiopulmonary bypass, postoperative management, and nursing. This iterative process ensured that the chosen features were relevant and reliable. All analyses were performed using two sets of features: the selected feature set (consisting of 19 features) and the full feature set (consisting of 31 features), which overlapped to some extent.

### Assessment of delirium

POD is a series of acute and fluctuating cognitive disturbances that commonly occur between postoperative days 2 and 5 after open-heart surgery [24]. Delirium assessment was performed twice daily by different paramedics in the CSICU for up to 7 days until a positive assessment result was obtained. Conversely, no change in mental state within seven days was considered a negative result. Patients who left the CSICU before the 7th day were assessed by trained paramedics in the wards.

First, the Richmond Assessment Sedation Scale (RASS score) was used to assess the sedation level of our patients. As previously described, patients with an RASS score of -4 (defined as comatose) or -5 (indicating no physical/verbal response) were excluded because they could not be screened using the Confusion Assessment Method (CAM) [21, 25]. After initial screening, delirium was assessed using the Confusion Assessment Method (CAM), the most widely used standardized bedside diagnostic tool, which has been shown in previous studies to be highly sensitive (94–100%) and specific (90–95%) [26, 27]. Patients were defined as positive when the following required diagnostic components were present: (1) an acute change in mental status over some time; (2) a decrease in concentration; (3) a change in level of consciousness; and (4) confusion in thinking structure. As long as the patient had either feature 1 and 2 or feature 3 and 4, the diagnosis of delirium was definitive [27, 28].

### Machine learning algorithms and analysis

A total of 507 patients who met our inclusion criteria were analyzed retrospectively. All computations and analyses were performed using Python version (3.12.0) and databases in Python such as pandas [29], numpy [30], random [31], seaborn [32], matplotlib [32], and sklearn [33], were applied. The features we selected are the information categories that must be recorded in the admission medical records and the routine monitoring items after entering the cardiac surgery ICU. To ensure the completeness of the data, we removed patients with a high proportion of missing data. The overall data missing rate is 0.18%. Among all variables, the height feature has the highest missing rate, accounting for 0.10% of the total sample size (Patients admitted to wheelchairs or stretchers often have missing data). For most of the missing data, we used the mode-filling method for processing. For the height and weight features, which have obvious differences between males and females, we calculated the mean weight for different genders and filled in the missing values according to the patient’s gender [34].

We employ the StratifiedShuffleSplit method to partition the dataset into two segments, a training set (80%, *N* = 405) and a validation set (20%, *N* = 103). The stratified sampling technique guarantees that the distribution of classes in both the training and test sets closely resembles that of the entire dataset [14, 35]. The random seed is set to 1 to ensure the reproducibility of the results. Normalization is carried out to eliminate variations in feature values, thereby improving the stability and dependability of the model. To ensure data privacy and prevent information leakage, a clear separation between the training and test sets is established before normalization. By employing the statistical properties derived solely from the training set, normalization is performed on the training set. Subsequently, the same transformation approach is applied to normalize the test set, ensuring consistent treatment across both datasets [36].

We applied several supervised machine learning methods to both the full and selected feature sets to construct predictive models of delirium, Specifically, we utilized classical machine learning algorithms commonly employed for classification problems, including Random Forest Classifier [37], Logistic Regression [38, 39], Support Vector Machine Classifier (SVC) [40], K-nearest Neighbors Classifier [41], Gaussian Naive Bayes [42], Gradient Boosting Decision Tree [43] and Perceptron [44]. The selection of these algorithms was determined by factors such as the sample size and number of features.

To better evaluate the model’s performance, we generated confusion matrices for each model on the training and testing sets. From these matrices, we calculated the accuracy (ACC), precision, recall, and F1-score for the full feature and selected feature datasets. Additionally, we used bootstrap sampling to estimate the area under the curve (AUC) and average precision (AP), along with their confidence intervals, for both the training and testing sets. This approach provided further insight into the stability and reliability of the models.

To enhance the interpretability of the machine learning model predictions and understand the relative importance of features in predicting outcomes, we used SHAP (Shapley Additive Explanations). Based on the Shapley value concept in cooperative game theory, this method allowed us to assess the contribution of each feature to the prediction results, and to identify any interactions between features. Furthermore, SHAP enabled us to explain individual sample behavior and overall model performance [45]. To quantitatively measure the calibration performance of a classification model, we utilize a metric called Expected Calibration Error (ECE). ECE calculates the average difference between predicted probabilities and the corresponding empirical probabilities, indicating the model’s calibration. By computing the ECE, we obtain a numerical value that represents the model’s calibration, enabling us to gain a better understanding and explanation of the model’s predictive quality [46].

## RESULT

### Participants characteristics

A brief description of the overall steps of this study is provided in Fig. 1. A total of 507 patients who met all inclusion criteria were enrolled between 30th June 2019 and 30th June 2020. According to the grouping methods employed in previous research, the enrolled cases were randomly allocated into a training group (80%, *N* = 405) and a validation group (20%, *N* = 103) [14]. Table 1 displays the baseline data for both the full feature set and the selected feature set. Continuous variables are presented as mean and standard deviation (SD), while categorical variables are presented as percentages.

**Table 1:** Patient Characteristics (Preoperative, Intraoperative, and Postoperative)

Patient characteristic	Total sampl(*N* = 507)	Full Feature Set (q = 31)		Selected Feature Set (q = 31)		Units
		Training data(*N* = 405)	Test data(*N* = 102)	Training data(*N* = 405)	Test data(*N* = 102)	
Delirium ( N (%) )	141 (28%)	113 (28%)	28(27%)	112 (28%)	29(28%)	
**Preoperative information**						
Female Sex ( N (%) )	300 (59%)	235 (58%)	65(63%)	235 (58%)	65(63%)	
Education score (mean (SD)*	0.5 (0.8)	0.5 (0.8)	0.5 (0.7)	0.5 (0.8)	0.5 (0.7)	Point
Age (mean (SD))	55.7 (13.5)	55.4 (13.6)	56.7 (13.0)	55.4 (13.7)	56.7 (13.0)	Years
Height (mean (SD))	157.1 (29.6)	156.9 (29.8)	157.5 (28.8)	157.0 (29.8)	157.5 (28.8)	Cm
Weight (mean (SD))	58.2 (11.3)	58.0 (11.1)	58.7 (11.7)	58.0 (11.1)	58.7 (11.7)	Kg
Alcohol abuse ( N (%) )	29 (6%)	20 (5%)	9(9%)	20 (5%)	9(9%)	
Smoke abuse ( N (%) )	84 (17%)	66 (16%)	18 (18%)	66 (16%)	18 (18%)	
Coronary heart disease ( N (%) )	34 (7%)	29 (7%)	5 (5%)			
Cerebral infarction ( N (%) )	33 (7%)	21(5%)	5(5%)			
Diabetes ( N (%) )	29 (6%)	21 (5%)	8 (8%)			
Hypertension ( N (%) )	87 (17%)	71 (15%)	16 (16%)			
LVEF (mean (SD)	58.7 (10.7)	58.8 (11.9)	57.6 (12.8)	58.9 (10.1)	57.6 (12.8)	%
**Intraoperative information**						
CPB duration (mean (SD))	157.3 (73.5)	158.3 (71.0)	153.1 (82.5)	158.3 (71.0)	153.1 (82.5)	Min
ACC duration (mean (SD))	97.9 (44.1)	97.6 (43.0)	96.7 (48.3)	98.2 (43.0)	96.7 (48.3)	Min
Anesthesia duration (mean (SD))	258.6 (100.5)	260.7 (101.6)	250.2 (96.3)			Min
**Postoperative information**						
IABP employ ( N (%) )	66 (13%)	51 (13%)	15 (15%)	51 (13%)	15 (15%)	
ECMO employ ( N (%) )	39 (8%)	30 (7%)	6 (6%)			
WBC (mean (SD))	14.1 (5.7)	14.2(5.9)	13.7 (5.0)			10^9^/L
NEUT (mean (SD))	11.7 (5.1)	11.8(5.3)	11.4 (4.5)			10^9^/L
LY (mean (SD))	1.4 (0.9)	1.4 (1.0)	1.4 (0.9)	1.4 (1.0)	1.4 (0.9)	10^9^/L
BUN (mean (SD))	8.7 (4.9)	8.6 (5.0)	8.7 (4.2)	8.8 (5.0)	8.7 (4.2)	mmol/L
TBLL (mean (SD))	25.3 (20.9)	24.9 (21.3)	24.0 (19.1)	25.6 (21.2)	24.0 (19.1)	mmol/L
Serum creatinine (mean (SD))	106.4 (92.4)	108.6 (99.9)	97.3 (52.2)	108.6 (99.9)	97.3 (52.2)	umol/L
Serum albumin (mean (SD))	35.4 (22.6)	35.7(25.0)	33.7(6.8)	35.8(25.0)	33.7(6.8)	g/L
PH (mean (SD))	7.3 (0.8)	7.3 (0.7)	7.3 (1.0)	7.3 (0.7)	7.3 (1.0)	
PaCO_2_ (mean (SD))	38.5 (15.1)	38.5 (16.3)	38.0 (8.6)	38.6 (16.3)	38.0 (8.6)	mmHg
PaO_2_ (mean (SD))	249.0 (102.9)	249.8 (102.8)	245.5 (103.5)			mmHg
Na (mean (SD))	139.1 (18.9)	139.58 (18.7)	134.4 (20.0)			mmol/L
K (mean (SD))	3.9 (0.7)	3.9 (0.7)	3.9 (0.7)			mmol/L
Glu (mean (SD))	9.1 (3.7)	9.1 (3.6)	8.9 (3.9)			mmol/L
Pain score (mean (SD))*	2.2 (0.9)	2.2 (0.9)	2.2 (1.0)	9.1 (3.6)	8.9 (3.9)	point

Data are presented as mean and standard deviation (SD) for continuous variables and as percentages for dichotomous variables. LVEF left ventricular ejection fraction, CPB Cardiopulmonary Bypass, ACC aortic cross-clamping, IABP Intra-Aortic Balloon Pump, ECMO Extracorporeal Membrane Oxygenation, WBC White Blood Cell Count, NEUT Neutrophils Count, LY Lymphocytes Count, BUN Blood Urea Nitrogen, TBLL Total Bilirubin

*Education score, range 0–2. Lower scores indicate lower educational attainment levels

*Pain score, range 0–10. Higher scores indicate greater pain levels

The incidence of delirium, which reached up to 28% in our study, suggested that an accurate predictive model for patients with VHD was essential. As for the assessment of educational attainment, we developed a scoring scheme by converting multi-categorical variables into continuous variables. According to our admission scoring system, the educational level of patients was divided into three categories: junior high school education and below (score as 0); high school education or undergraduate degree (score as 1); postgraduate degree and above (score as 2). The average education score of the overall samples was 0.5, indicating a low level of education among our participants. Some researchers considered low educational level as a risk factor for the development of delirium due to the lack of mental training activities and insufficient cognitive reserve [47, 48].

The goal of this study is to predict the risk of developing delirium within 24 h of admission to the CSICU and to take preventive measures in the early stages of the disease. Referring to the diagnostic and treatment practices for postoperative admission to the CSICU, the laboratory test results of patients are obtained within 24 h of admission. The earliest results within 24 h are used for machine learning and model training purposes. In addition to the conventional indicators of postoperative laboratory tests, the postoperative use of IABP/ECMO within the delirium assessment period was also included in this study. IABP/ECMO is associated with hemodynamic instability and internal environmental disturbances, which may lead to the development of delirium [49–51]. In this study, we included the pain score assessed by the Digital Evaluation Scale (NRS) as a predictor of delirium outcome [52].

### Model performance

We utilized the following machine learning methods to develop prediction models for POD in patients with VHD, respectively using full variables and selected variables as input features: Random Forest Classifier [37], Logistic Regression [38, 39], SVC [40], K-nearest Neighbors Classifier [41], Gaussian Naive Bayes [42], Gradient Boosting Decision Tree [43] and Perceptron [44]. These algorithmic models were then validated in the validation group to assess their performance.

**Fig. 2:**
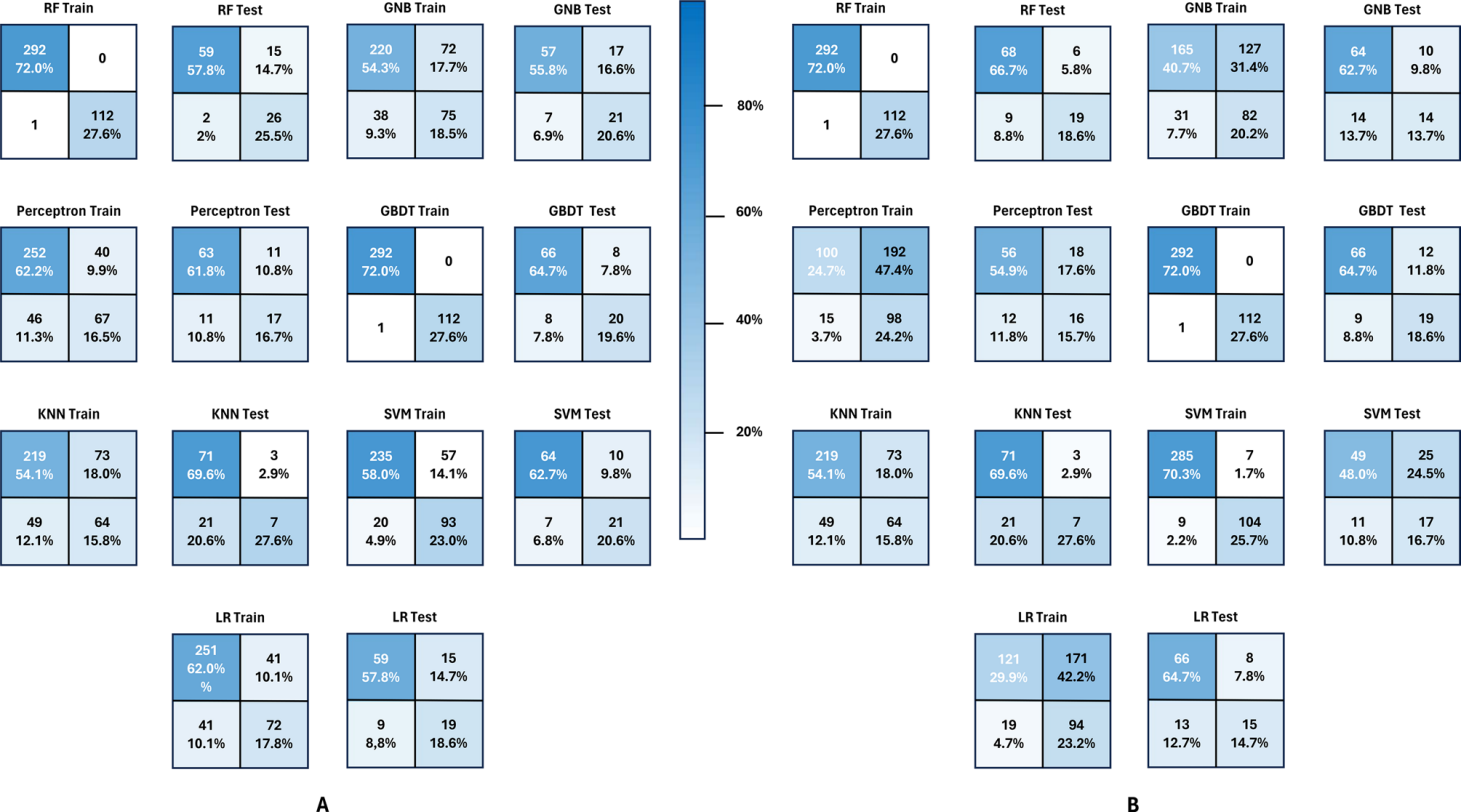
Comparison of Confusion Matrixs among machine learning models under full feature set (**A**) and selected feature set (**B**). The confusion matrix is a 2 × 2 matrix that contains the following four elements: True Positive (top left of the matrix); False Positive (bottom left of the matrix); True Negative (bottom right of the matrix); False Negative (top right of the matrix). Through the analysis of the confusion matrix, we can calculate many performance indicators such as accuracy, recall, precision, and F1 score to evaluate the classification performance of the model on different categories

To gain a better understanding of the performance of the models, we plotted the confusion matrices for each model on both the training and testing datasets. The confusion matrix based on the full feature data is summarized in Fig. 2A, while the confusion matrix based on the selected feature data is summarized in Fig. 2B. In the confusion matrix, the top-left element represents true positives, the top-right element represents false positives, the bottom-left element represents false negatives, and the bottom-right element represents true negatives. Based on the computational results, we obtained the accuracy (ACC), Precision, Recall, and F1 score for the models using both the full feature and selected feature datasets. Additionally, we reported the AUC and AP of seven machine learning algorithms based on both the full feature and selected feature data. We also reported the ECE for each algorithm and summarized the above indicators in Table 2. Our model, based on the full feature set, performed well. To further assess its performance across different populations, we divided the full feature set into five additional test subsets: age ≥ 65, BMI ≤ 18.5, body mass index > 28, history of stroke, and history of coronary heart disease. These subsets were chosen based on the impact of old age [28], physical weakness [53], metabolic disorders [54], history of Cerebral infarction [55], and history of coronary heart disease [56] on the outcome of delirium. To evaluate its calibration performance in different scenarios, we separately calculated the model’s ECE on each subset and summarized it in Table 3.

**Table 2:** Comparison of Machine Learning Algorithms for Prediction of Delirium in Two Overlapping Feature Sets (Ranking based on AUC)

Algorithms	Accuracy	Precision	Recall	F1	AUC (95% CI)	AP (95% CI)	ECE
**Full feature set (q = 31)**							
Random Forest Classifier	0.83	0.80	0.86	0.81	0.92 (0.91–0.92)	0.80 (0.78–0.82)	0.10
Gradient Boosting Decision Tree	0.84	0.80	0.80	0.80	0.90 (0.89–0.91)	0.79 (0.76–0.81)	0.05
Support Vector Machine Classifier	0.83	0.79	0.81	0.80	0.83 (0.81–0.84)	0.68 (0.66–0.71)	0.15
Logistic Regression	0.76	0.71	0.74	0.72	0.80 (0.78–0.82)	0.64 (0.61–0.67)	0.06
Gaussian Naive Bayes	0.76	0.72	0.76	0.73	0.79 (0.77–0.80)	0.57 (0.55–0.59)	0.08
K-nearest Neighbors Classifier	0.76	0.74	0.60	0.61	0.78 (0.76–0.80)	0.55 (0.52–0.58)	0.14
Perceptron	0.78	0.73	0.73	0.73	0.77 (0.75–0.78)	0.60 (0.57–0.62)	0.23
**Selected feature set (q = 19)**							
Random Forest Classifier	0.78	0.73	0.73	0.73	0.86 (0.85–0.88)	0.73 (0.70–0.75)	0.14
Gradient Boosting Decision Tree	0.76	0.74	0.60	0.61	0.83 (0.82–0.84)	0.61 (0.59–0.64)	0.13
Logistic Regression	0.83	0.80	0.86	0.81	0.76 (0.74–0.77)	0.61 (0.58–0.63)	0.13
K-nearest Neighbors Classifier	0.83	0.79	0.81	0.80	0.72 (0.70–0.74)	0.50 (0.47–0.53)	0.11
Gaussian Naive Bayes	0.76	0.71	0.74	0.72	0.70 (0.68–0.72)	0.50 (0.48–0.53)	0.12
Perceptron	0.76	0.72	0.76	0.73	0.69 (0.67–0.71)	0.50 (0.47–0.54)	0.31
Support Vector Machine Classifier	0.84	0.80	0.80	0.80	0.64 (0.62–0.67)	0.52 (0.49–0.55)	0.14

Accuracy= (TP + TN) / (TP + FP + TN + FN) ; Precision = TP / (TP + FP) ; Recall = TP / (TP + FN) ; F1 Score = 2 * (Precision * Recall) / (Precision + Recall) ; TP = true positive ; TN = true negative ; FP = false positive ; FN = false negative

AUC is the area under the receiver operating characteristic curve; AP is the average Precision; ECE is the expected Calibration Error

**Table 3:** ECE in the test dataset and the 5 specific population subsets

ECE	Test dataset (*n* = 102)	Age ≥ 65 (*n* = 131)	BMI ≤ 18.5 (*n* = 64)	BMI>28 (*n* = 29)	Cerebral infarction (*n* = 33)	Coronary heart disease (*n* = 34)
**Algorithms**						
Random Forest Classifier	0.10	0.13	0.16	0.14	0.19	0.21
Gradient Boosting Decision Tree	0.05	0.02	0.01	0.07	0.05	0.08
Support Vector Machine Classifier	0.15	0.28	0.30	0.29	0.32	0.21
Logistic Regression	0.06	0.06	0.11	0.13	0.13	0.04
Gaussian Naive Bayes	0.08	0.14	0.13	0.14	0.08	0.21
K-nearest Neighbors Classifier	0.14	0.06	0.04	0.15	0.21	0.24
Perceptron	0.23	0.20	0.17	0.21	0.24	0.14

The test dataset includes all features (*n* = 102)

Specific population subset 1: Patients aged ≥ 65 (*n* = 131); Specific population subset 2: Patients with BMI ≤ 18.5 (*n* = 64); Specific population subset 3: Patients with BMI > 28 (*n* = 29); Specific population subset 4: Patients with a history of cerebral infarction (*n* = 33); Specific population subset 5: Patients with a history of coronary heart disease (*n* = 34)

**Fig. 3:**
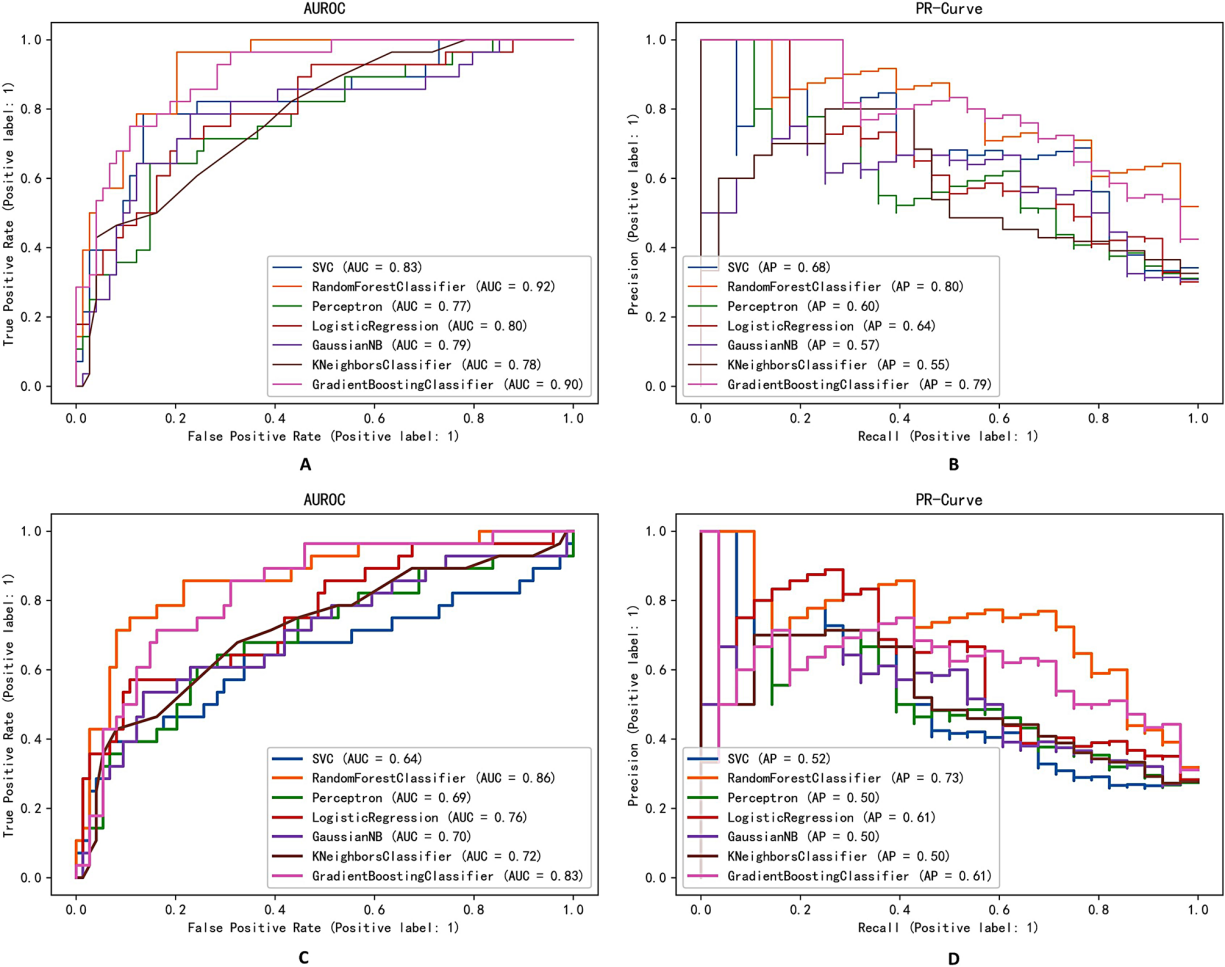
Comparison of AUROCs among machine learning models under full feature set (**A**) and selected feature set (**C**). Comparison of PR-Curves among machine learning models under full feature set (**B**) and selected feature set (**D**). Random Forest Classifier yielded the greatest AUC and AP in both feature set

AUROC (Area Under the Receiver Operating Characteristic Curve) for prediction models, generated by plotting the true positive rate (TPR) against the false positive rate (FPR) at various threshold settings, are shown in Fig. 3A (full feature set) and Fig. 3C (selected feature set), respectively [57]. The AUC is a metric that ranges from 0 to 1, where a value of 1 represents a perfect classifier and a value of 0.5 suggests random predictions. The Random Forest Classifier exhibits excellent performance in both feature datasets, with an AUC of 0.92 (95% CI, 0.91–0.92) for the full feature dataset and an AUC of 0.86 (95% CI, 0.85–0.88) for the selected feature dataset. Similarly, the Gradient Boosting Decision Tree exhibits relatively strong predictive performance, with an AUC of 0.90 (95% CI, 0.89–0.91) for the full feature dataset and an AUC of 0.83 (95% CI, 0.82–0.84) for the selected feature dataset. The classical algorithm Logistic Regression consistently performs well, yielding an AUC of 0.80 (95% CI, 0.78–0.82) for the full feature dataset and an AUC of 0.76 (95% CI, 0.74–0.77) for the selected feature dataset. In contrast, the SVC shows relatively unstable performance, with an AUC of 0.83 (95% CI, 0.81–0.84) for the full feature dataset and an AUC of 0.64 (95% CI, 0.62–0.67) for the selected feature dataset. All algorithms achieve a higher AUC in the full feature set compared to the selected feature set.

The PR-Curve is a graphical representation of the precision and recall relationship of a classifier at different thresholds, as shown in Fig. 3B (full feature set) and Fig. 3D (selected feature set). In the PR-Curve plot, the horizontal axis represents the recall, which is the proportion of true positive samples correctly identified by the model out of all actual positive samples. The vertical axis represents the precision, which is the proportion of true positive samples among the predicted positive samples. When the threshold is set to 0, it means that the model classifies all samples as positive instances. As a result, the recall for this model will be 1 because it can correctly identify all true positive instances. However, since the model classifies all samples as positive, there may be some negative instances (true negatives) that are falsely classified as positive (false positives). This leads to a decrease in precision, which approaches but does not exactly equal 0 [58]. From the graphs, it can be observed that the PR-Curve curves of the Random Forest Classifier for both full feature datasets are closer to the top-right corner compared to other curves. The Random Forest Classifier also achieves the highest AP, 0.80 (95% CI, 0.78–0.82) in the full feature dataset and 0.73 (95% CI, 0.70–0.75) for the selected feature dataset. Although the PR-Curve curve of the Gradient Boosting Decision Tree is similar to that of the Random Forest Classifier for the full feature dataset, it exhibits significant fluctuations for the selected feature dataset, resulting in a lower AP of 0.61 (95% CI, 0.59–0.64). Overall, all models perform better in terms of AP on the full feature dataset compared to the selected feature dataset.

**Fig. 4:**
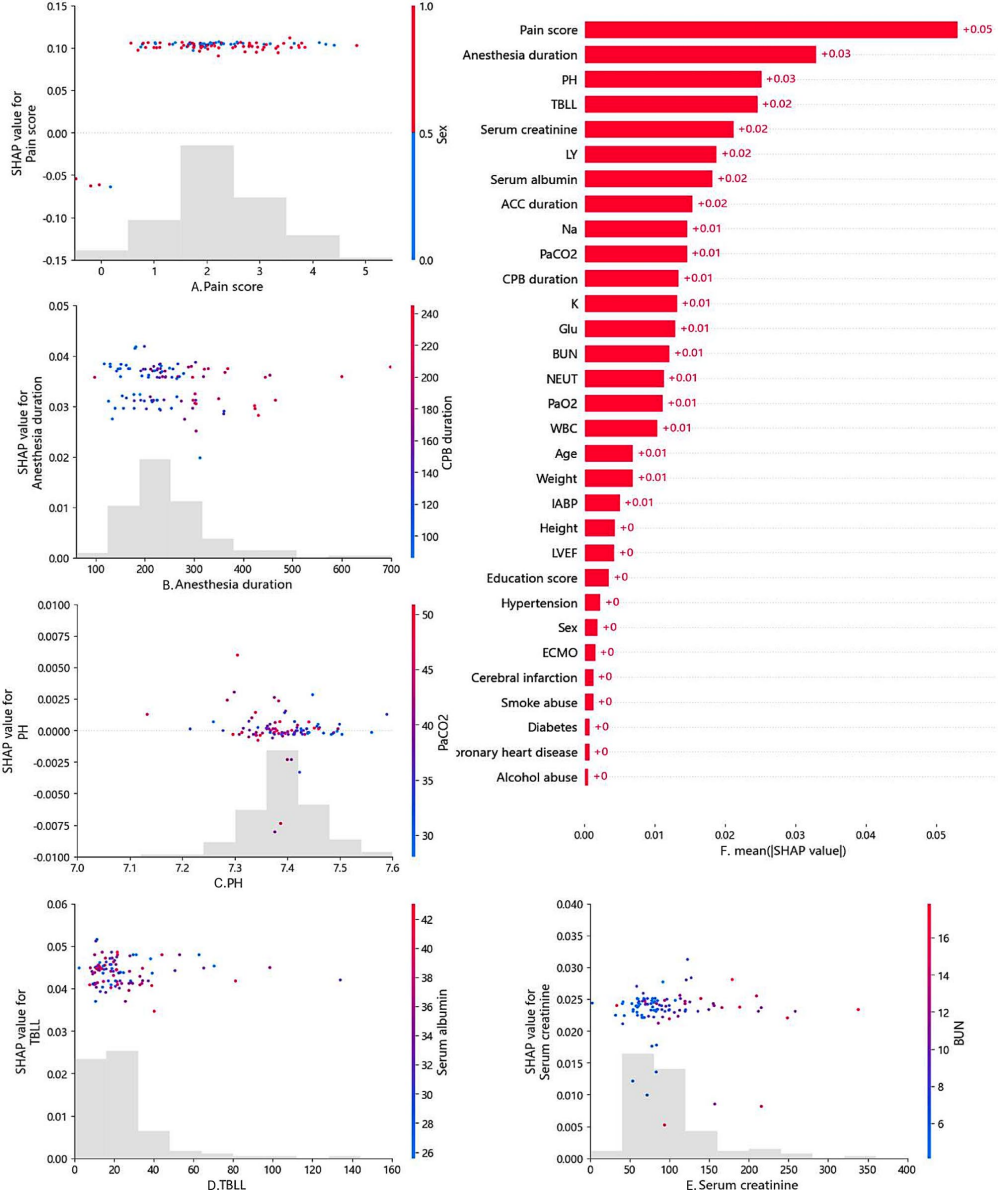
Scatter plot of pain score colored by sex (**A**). Scatter plot of anesthesia duration colored by CPB duration(**B**). Scatter plot of PH colored by PaCO2 (**C**). Scatter plot of TBLL colored by serum albumin (**D**). Scatter plot of serum creatinine colored by BUN (**E**). The SHAP importance matrix plots of Random Forest Classifier under full feature set (**F**)

**Fig. 5:**
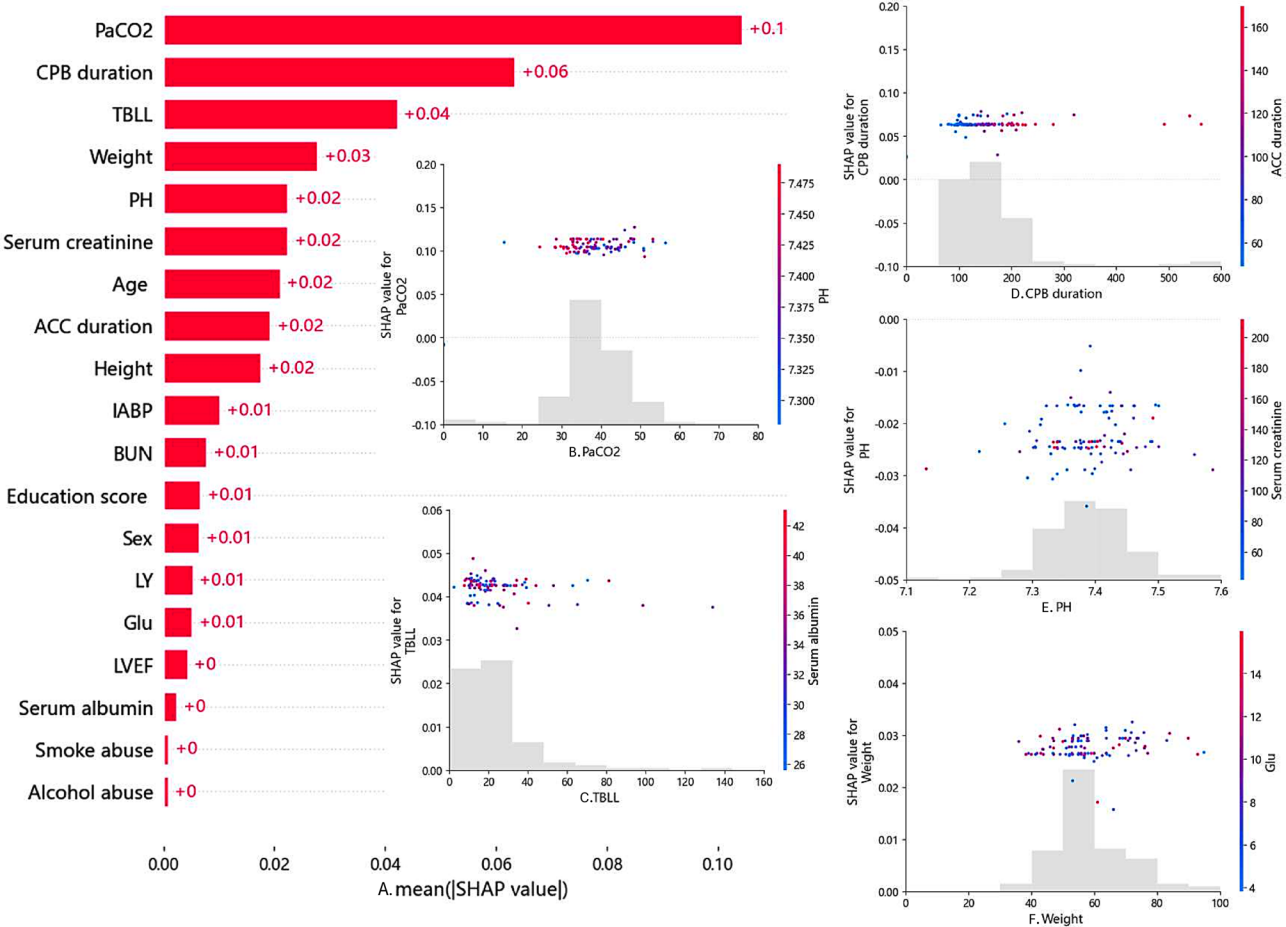
The SHAP importance matrix plots of Random Forest Classifier under selected feature set (**A**). Scatter plot of PaCO2 colored by PH (**B**). Scatter plot of TBLL colored by serum albumin (**C**). Scatter plot of CPB duration colored by ACC duration (**D**). Scatter plot of PH colored by serum creatinine (**E**). Scatter plot of Weight colored by Glu (**F**)

Further evaluation of the best-performing Random Forest Classifier was carried out using SHAP (Shapley Additive Explanations), which is a method for explaining the predictions of machine learning models. Based on the Shapley value concept in cooperative game theory, it aims to provide a measure of contribution to the prediction result for each feature. SHAP values for specific features exceeding zero represent an increased risk of POD development [45]. The importance matrix plot of the Random Forest Classifier is shown in Fig. 4F (full feature set) and Fig. 5A (selected feature set). We have also generated SHAP scatter plots for the top five ranked features in the full feature dataset (Fig. 4A-E) and selected feature dataset (Fig. 4B-F), and colored them based on their potential correlating factors [59].

To determine the features that have the greatest impact on the random forest prediction model, we plotted SHAP summary plots for two feature datasets. The top 20 features in the full feature set (Fig. 5A) and all features in the selected feature set (Fig. 5B) are shown in the plot. This figure describes the degree of relative importance of feature values in the training dataset concerning the SHAP value. The higher the SHAP value of a feature, the greater the likelihood of delirium occurrence. We also summarized the ECE in Table 2 to quantitatively measure the calibration performance of the classification models. Except for the SVC, the other prediction models showed lower ECE values in the full feature set compared to the selected feature set.

## DISCUSSION

We developed the first machine learning-based prediction model for POD outcomes in patients with VHD. The incidence of delirium in VHD patients after valve surgery can reach up to 28% (*n* = 141), necessitating a method to predict POD and aid in clinical prevention. Compared to previous predictive models for POD after cardiac surgery, this study explicitly focuses on the POD of VHD patients in the ICU, taking advantage of the large number of valve surgeries performed annually and the presence of the CSICU in our hospital. Furthermore, all participants underwent elective valve surgery with cardiopulmonary bypass [15]. We mainly set a period of seven days to assess delirium to increase the rigor, because POD is an acute postoperative syndrome that usually develops 2–5 days after surgery. This differs from the later delirium, which may be seen in longer ICU stays, and is often more complex in its causes of morbidity and therefore worthy of separate discussion. Many previous studies on prediction models either ignore the setting of a prediction time point or have problems with the interval between the prediction time point and the outcome [24]. We developed a POD prediction model for VHD patients admitted to the CSICU with valve surgery. The predictive time point is set within 24 h of ICU admission, allowing us to perform risk assessment and outcome prediction based on preoperative assessments, intraoperative information, and postoperative initial laboratory results. Care plans and treatments are then adjusted to address the higher risk of delirium.

The characteristics of our participants were associated with the surgical procedure and the pathogenetic features of VHD. For example, our participants were predominantly female (*n* = 300, 59%; Table 1), and the average age (55.7 years, SD 13.5; Table 1) was below 70 years, as VHD is more common in females and middle-aged individuals, which differs from other models that do not differentiate the primary disease [15]. The average cardiopulmonary bypass (CPB) duration (157.3 min (SD 73.5; Table 1) versus 198.34 min), aortic cross-clamping (ACC) duration (97.9 min (SD 44.1; Table 1) versus 114.86 min), and anesthesia duration (258.6 min (SD 100.5; Table 1) versus 476.91 min) were much shorter in this study compared with the previous POD study in patients with type A aortic dissection (AAD) [60]. The average pain score of 2.2 points (SD 0.9; Table 1) in this study indicates mild pain after surgery. Poor pain management caused by inadequate analgesia or excessive sedation may trigger delirium after surgery [61].

Interest in using machine learning algorithms for risk assessment and clinical outcome prediction has grown due to the advancement of artificial intelligence (AI) software and the reliability of AI algorithms. In this study, machine learning applications are used to create an efficient prediction model (Fig. 1). The Random Forest Classifier, a common machine learning technique, outperforms other current algorithms in terms of accuracy and produces an internal estimate of its generalization error during training [62]. In this retrospective study, the Random Forest Classifier with a full feature set achieved an AUC of 0.92 (95% CI, 0.91–0.92; Fig. 3A; Table 2), indicating excellent performance in delirium prediction. Even with a simpler feature set, the Random Forest Classifier achieved an AUC of 0.86 (95% CI, 0.85–0.88; Fig. 3C; Table 2), providing a relatively high predictive value, allowing it to be used for initial screening in some situations. In addition to achieving a high AUC, the Random Forest Classifier also achieved the highest AP, with an AP of 0.80 (95% CI, 0.78–0.82; Fig. 3C; Table 2) in the full feature dataset and an AP of 0.73 (95% CI, 0.70–0.75; Fig. 3D; Table 2) in the selected feature dataset. The larger the area under the PR curve (i.e., the greater the AP value), the better the performance of the classification model, indicating that the model can maintain sufficient accuracy while maintaining a high recall rate. In practical applications, it’s common for the predicted probabilities of a model to not perfectly align with the true observed frequencies, necessitating calibration. The ECE of the Random Forest Classifier is relatively small as well (0.1 for the full feature set and 0.14 for the selected feature set; Table 2), which indicates that the model has good consistency between its predicted probabilities and the observed outcomes, showcasing excellent potential for practical applications. The above indicators all point out the outstanding predictive ability of the Random Forest Classifier. Furthermore, when compared to neural networks, a widely utilized method known for its exceptional information processing capability, the Random Forest Classifier yields satisfactory results with a significantly smaller sample size [62]. This makes it a potentially exciting alternative for the future.

**Fig. 6:**
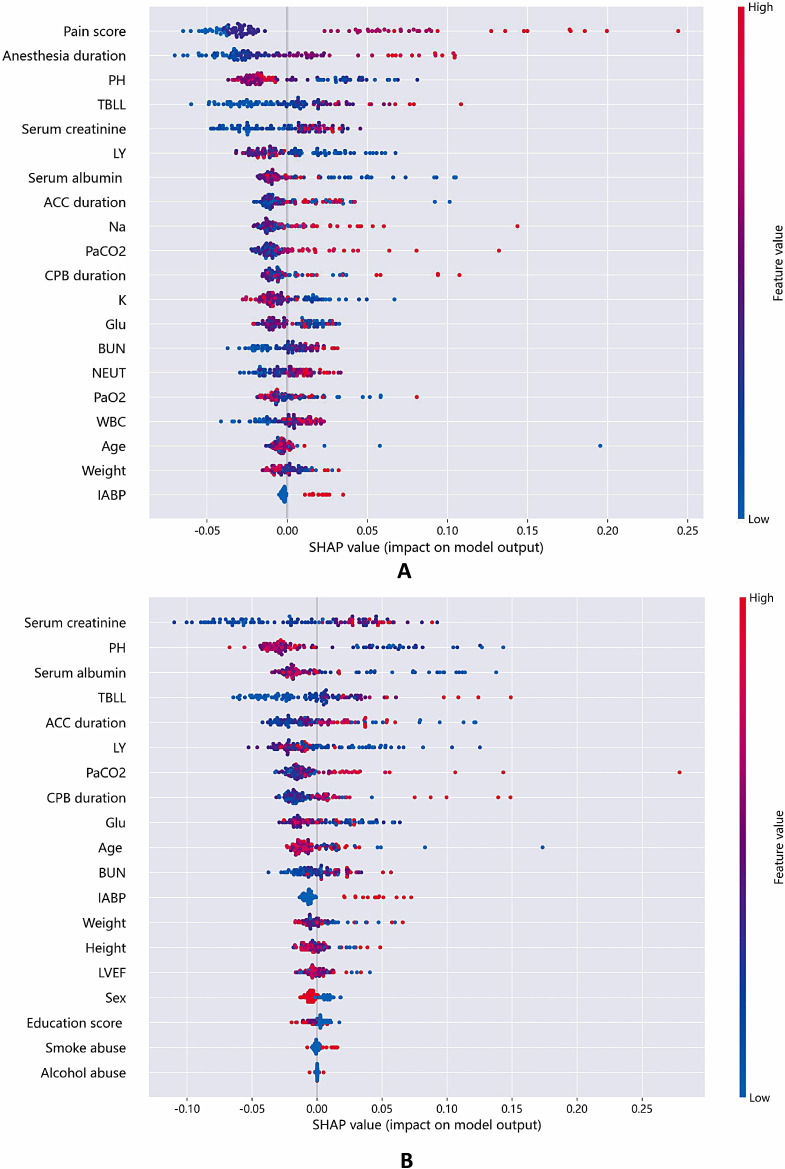
The SHAP summary plots of Random Forest Classifier under full feature set (**A**) and selected feature set (**B**). The higher the SHAP value of the feature, the higher the probability of delirium after surgery. A point is created for each attribution value of each feature of the model for each patient. Therefore, a point is assigned to each row of features for a patient. The points are colored based on the corresponding patient’s feature value and vertically accumulated to depict density. Red represents higher feature values, and blue represents lower feature values

In the test dataset covering all features, the ECE of the Random Forest Classifier model is 0.10. The difference in ECE compared to the test dataset is relatively small in subsets involving populations aged ≥ 65, BMI ≤ 18.5, and body mass index > 28. However, in subsets related to populations with a history of stroke and a history of coronary heart disease, the difference in ECE is relatively large, both exceeding 0.15, indicating a decrease in the model’s predictive ability in these specific subsets. Given the small sample size in these categories, this may lead to inaccurate predictions by the model. Increasing the sample size in future research could enhance the model’s performance in these subsets. Additionally, we observed that the ECE of the Gradient Boosting Decision Tree is very low in the test dataset covering all features (0.05), and similar in most feature subsets. In contrast, the Perceptron algorithm exhibits high ECE in both the validation set and the 5 specific population subsets, suggesting relatively poor calibration performance. These insights are expected to guide algorithm selection in future related research.

The prediction models used depend on the primary disease, population characteristics, and type of surgery, which may help improve the management and prevention of POD. Refining our delirium prediction models is an important part of advancing the field. This study explored the combination of machine learning and clinical applications, comparing different classifiers to verify that the Random Forest Classifier is a reliable approach to building prediction models, potentially providing a reference for research with a small sample size.

In this study, we utilized SHAP to analyze the prediction results of the Random Forest Classifier. In our SHAP Importance matrix plot, we can observe the ranking of importance and specific contribution values for all variables in the two feature datasets. In the full feature dataset, the top 5 most important variables that affect the model are pain score, anesthesia duration, PH, TBLL, and serum creatinine (Fig. 4F). For the selected feature dataset, the top 5 most important variables that affect the model are serum creatinine, PH, serum albumin, TBLL, and ACC duration(Fig. 5A). The SHAP value represents the contribution of a feature to the model’s predicted output, and it can be either positive or negative. A positive SHAP value indicates that the corresponding feature positively contributes to increasing the predicted value. In other words, when the feature takes a higher value, it amplifies the model’s prediction for a certain category or numerical value. Conversely, a negative SHAP value indicates that the corresponding feature negatively contributes to decreasing the predicted value.

We separately plotted scatter plots for the top five ranked features in the full feature dataset and selected feature datasets and colored them based on their potential correlating factors. For example, in the scatter plot of Painscore (Fig. 4A), the horizontal axis represents the value of Painscore for each sample, the vertical axis represents its SHAP value, and the coloring reflects the gender of each sample. Similarly, the horizontal axes of the corresponding plots in the other four subfigures represent the values of anesthesia duration, PH, TBLL, and serum creatinine for each sample (Fig. 4B-E), while the vertical axis represents the SHAP value of each feature, and the coloring reflects the values of CPB duration, PaCO2, serum albumin, and BUN for each sample (Fig. 4B-F). In the five subplots of the selected feature dataset, the horizontal axes represent the values of PaCO2, TBLL, CPB duration, PH, and weight of each sample (Fig. 5B-F), while the vertical axis represents the SHAP value of each feature, and the coloring reflects the values of PH, serum albumin, ACC duration, serum creatinine, and blood glucose for each sample (Fig. 5B-E).

In the full feature dataset, features such as Painscore, anesthesia duration, PH, TBLL, and serum creatinine rank high in the SHAP score, indicating that they have a great impact on the model’s predictive results. However, the overall SHAP values of the samples did not show a significant trend in the scatter plot. This suggests that each feature may have certain correlations or interactions with multiple other features, causing the impact of a single feature on the model output to be offset or masked by other features. Therefore, although they contribute significantly to the model’s predictive results overall, the specific values of these features have a relatively small impact on the model’s predictive results in individual sample analysis. This also confirms to some extent the complexity and multifactorial nature of postoperative delirium as a disease.

Finally, we summarized the SHAP summary plots of the full feature set (Fig. 6A) and the selected feature set (Fig. 6B). SHAP summary plots show the average SHAP value of each feature across all samples, providing an intuitive understanding of the overall patterns. They allow us to visually assess how each feature influences the overall prediction while observing the feature’s importance. Each row represents a feature, and the x-axis represents the SHAP value. Each point represents a sample, and the color represents the feature value (red indicates high values, and blue indicates low values).

Through the overall analysis of the images, among the top ten features in the full feature set, Pain score, Anesthesia duration, TBLL, Serum creatinine, ACC duration, Na, and PaCO2 show a positive correlation with the predicted value, while PH, LY, and Serum albumin show a negative correlation (Fig. 6A). This is consistent with previous research findings that inadequate postoperative pain management [61], prolonged anesthesia duration [63], electrolyte imbalance, metabolic abnormalities [64], abnormal liver and kidney function [3], carbon dioxide retention [65], monocyte-to-lymphocyte ratio (MLR) [66], and decreased serum albumin [67] can all contribute to the occurrence of postoperative delirium. By comparing the summary plots of the two feature sets and considering the previous outcome indicators, it can be observed that despite having only 19 features, the selected feature set still has meaningful predictive power for the outcome. Given its convenience in data collection, it can serve as a simple preliminary screening tool and be applied in clinical settings.

While clinical caregivers possess extensive bedside experience, their workload is often overwhelming. In the current medical environment, there is a critical shortage of medical personnel, necessitating urgent measures to alleviate their burden and allocate resources effectively. AI technology, with its powerful learning and computational capabilities, can play a significant role in addressing these challenges. Although there may be challenges during the initial implementation stages, continuous adjustments and optimizations of algorithms can enhance the efficiency of clinical work. It’s important to note that while AI cannot replace experienced clinical caregivers, it can augment their abilities by improving clinical decision-making and operational efficiency, while still preserving the indispensable role of skilled healthcare professionals at the bedside. Patients will benefit from the application of AI technology as it reduces the risk of human error caused by excessive clinical workload. Additionally, healthcare workers will be able to handle POD more easily and efficiently, resulting in improved patient care. If a patient is identified by an AI method as having a potential for POD, their care approach may differ from other patients. Clinicians may need to monitor them more closely and consider additional interventions to prevent or manage POD. This could involve adjusting the patient’s medication regimen or providing extra support for their mental and emotional well-being. The use of AI methods for identifying at-risk patients with POD has the potential to improve the quality of care and outcomes for these patients, while also enabling clinicians to make more informed decisions based on objective data.

Our study aims to advance AI healthcare by addressing the limitations of existing delirium prediction models. The algorithm developed in this study specifically targets patients undergoing valve surgery and incorporates routine data collected after admission to the CSICU. By integrating this algorithm into our medical information system, we can automatically retrieve patient data for analysis and conduct risk assessments based on the algorithm results. This enables adjustments to be made in medication and treatment decisions for high-risk patients. With the development of relevant software and the updating of ICU monitoring systems with well-trained prediction models, automatic alerts for delirium can be sent to clinicians in real-time, effectively reducing their workload.

There are limitations to this study. The sample size limits our choice of machine learning algorithms, and there was no external validation. We are considering increasing the sample size in future studies and performing external validation. The results and model parameters in this study will be helpful in our future research and provide a reference for similar studies.

## CONCLUSION

We developed the first POD prediction approach for CSICU-admitted VHD patients, which may be promising for automatically alerting ICU staff in the early stages of delirium. The Random Forest Classifier performs an AUC of 0.92 for the full feature dataset and an AUC of 0.86 for the selected feature dataset. Additionally, it achieves a relatively lower ECE and the highest AP, with an AP of 0.80 for the full feature dataset and an AP of 0.73 for the selected feature dataset. SHAP (Shapley Additive Explanations) was used to evaluate the Random Forest Classifier, and the importance matrix plot, scatter plots, and summary plots showcasing excellent potential for practical applications.

## Data Availability

The data supporting this study’s findings are available from the corresponding author (Prof. Liming LEI) upon reasonable request.
